# Septic pulmonary embolism arising from a small temporal boil in a patient with diabetes mellitus type 2: A rare case report

**DOI:** 10.1002/ccr3.8790

**Published:** 2024-05-10

**Authors:** Sanem Yildirim, Yavuz Yigit, Baha Hamdi Alkahlout, Eslam Hussein Mohamed, Aftab Mohammad Azad

**Affiliations:** ^1^ Department of Emergency Medicine Hamad Medical Corporation Doha Qatar; ^2^ Blizard Institute Queen Mary University London UK; ^3^ College of Medicine Qatar University Doha Qatar

**Keywords:** boil, cavitation, peripheral nodules, septic pulmonary embolism, Staphylococcus aureus

## Abstract

Septic pulmonary embolism (SPE) can originate from unusual sources like small boils, warranting consideration of diverse etiologies in respiratory distress. Prompt diagnosis, tailored antibiotics, and vigilant complication management optimize outcomes. Early recognition and treatment of minor infections, especially in diabetes are crucial.

## 1 INTRODUCTION

Septic pulmonary embolism (SPE) is a relatively rare form of nonthrombotic pulmonary embolism where infected clots originating from a primary infectious site travel to the pulmonary artery. This process results in infarctions and localized abscesses within the pulmonary vasculature. Traditionally, SPE has been linked to conditions such as infective endocarditis, intravenous drug use, oropharyngeal infections, and septic thrombophlebitis as seen in Lemierre syndrome.^1^

The clinical presentation of SPE can be nonspecific, making it a diagnostic challenge. Patients may experience symptoms such as fever, cough, pleuritic chest pain, and shortness of breath. These manifestations can overlap with those of respiratory or other conditions, further complicating the diagnostic process.^2^, ^3^ Early identification of such patients could lead to better results, fewer incorrect nonadmissions, and possibly shorter ICU stays.^4^

We present a remarkable case of SPE originating from a small boil on the right temporal region, an atypical source of infection. This case underscores the importance of considering rare diagnostic possibilities, especially in the context of underlying medical conditions. Additionally, it sheds light on the complexities of managing SPE and associated complications.

## 2 CASE HISTORY

**FIGURE 1:**
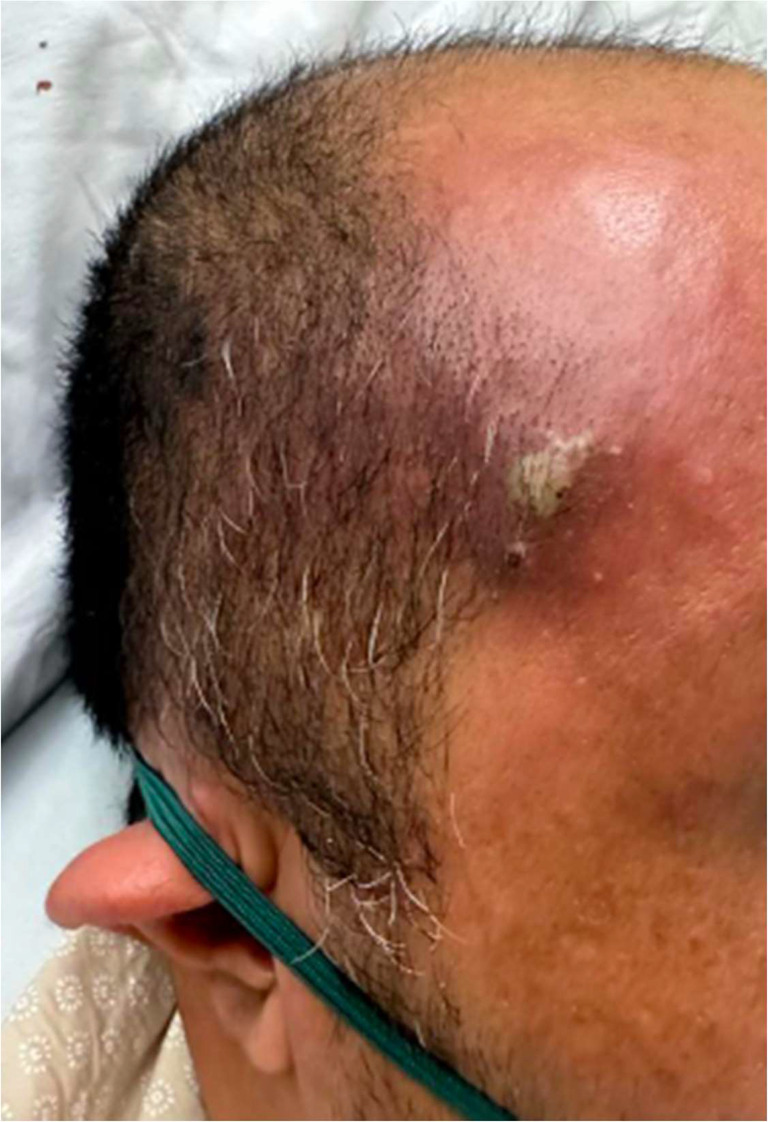
A boil situated in the patient's right temporal region.

A 44‐year‐old male with poorly controlled diabetes mellitus type 2 on metformin presented to the Emergency Department with a two‐day history of right‐sided chest pain and shortness of breath. Remarkably, the patient had a 1 cm boil on his forehead (Figure 1) with minimal pus formation, which he had noticed a few days earlier. Upon presentation, vital signs indicated hypotension, tachycardia, tachypnea, and fever consistent with sepsis: Blood Pressure 94/53 mmHg, Heart Rate 102 bpm, respiratory rate 26 breaths per minute, oxygen saturation 98%, temperature 38.2°C.

## 3 METHODS

Laboratory investigations revealed leukocytosis with a white blood cell count of 21.5 × 10^9^/L, elevated neutrophil count at 20.2 × 10^9^/L, elevated C‐reactive protein at 175 mg/L, impaired renal function with a creatinine level of 131 μmol/L, hyperglycemia with a random blood sugar level of 11.5 mmol/L, and elevated glycated hemoglobin (HbA1C) levels at 8%, suggestive of systemic inflammation and poorly controlled diabetes mellitus type 2.

Concurrently, he experienced symptoms of sepsis, including fever, chills, and generalized malaise. The patient's history of poorly controlled diabetes mellitus type 2 added a layer of complexity to the case, raising concerns about the potential impact of glycemic control on the course of infection.

Despite the modest size of the forehead boil, the patient exhibited severe respiratory distress and an elevated heart rate. Initial management included symptomatic treatment, pulmonary embolism prophylaxis, and broad‐spectrum antibiotics. Given the presentation's severity and rapid progression, a comprehensive diagnostic workup, including pulmonary embolism and septic workup, was initiated.

**FIGURE 2:**
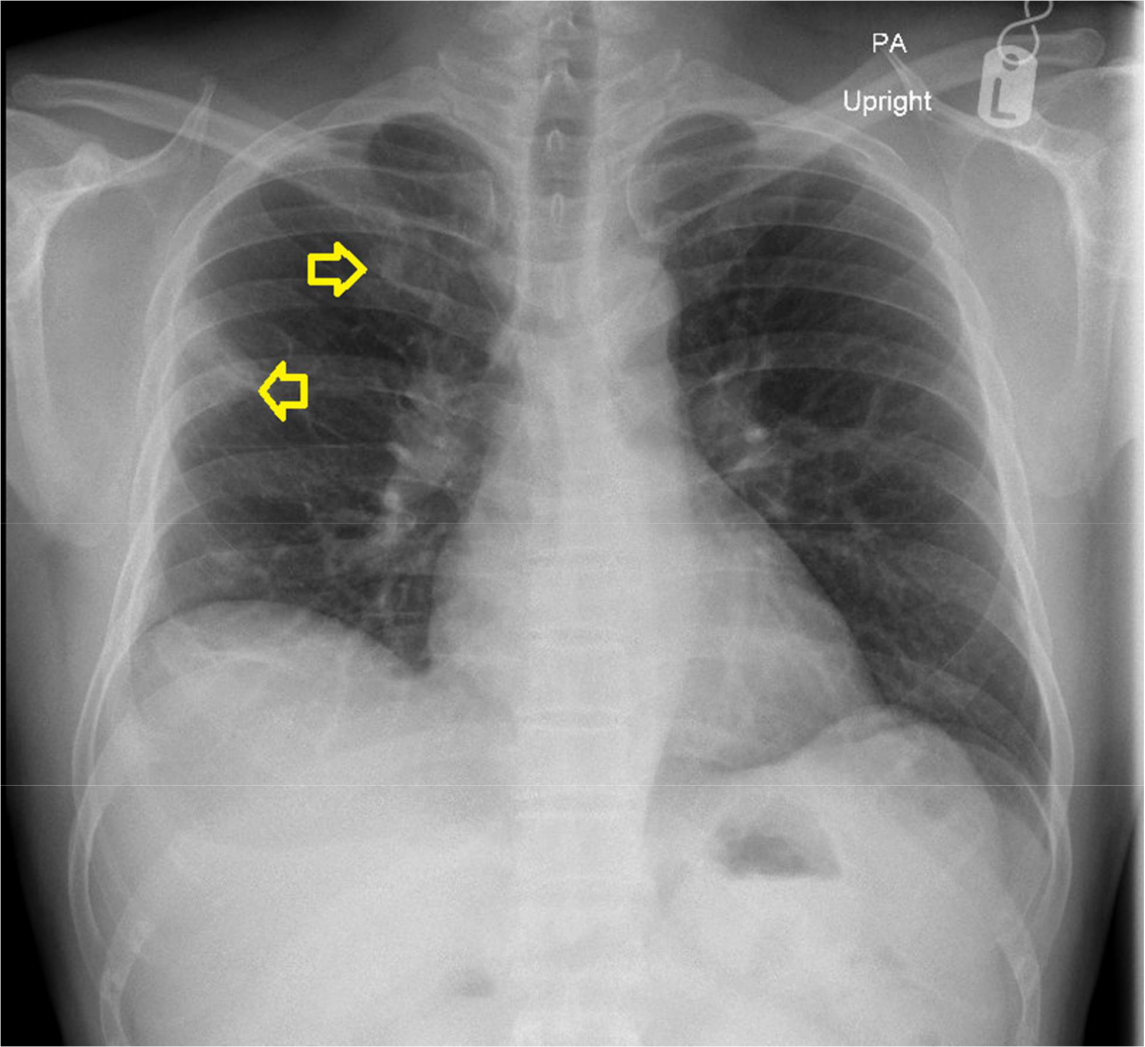
Multiple pulmonary opacities observed on chest X‐ray.

**FIGURE 3:**
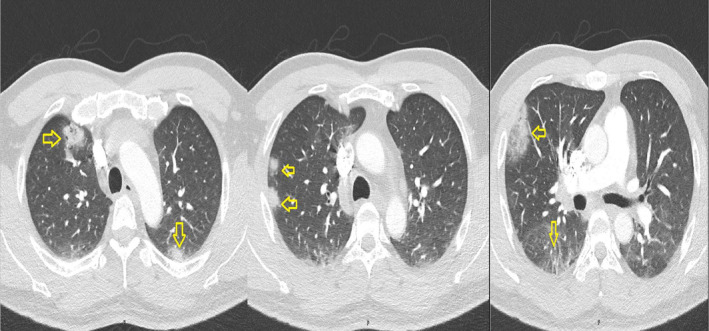
Wedge‐shaped lesions observed in both lungs.

Surprisingly, imaging studies revealed an unexpected finding on chest X‐ray—multiple lung opacities (Figure 2). A subsequent thoracic CT scan with contrast unveiled wedge‐shaped lesions in bilateral lungs. (Figure 3). These findings were consistent with SPE. Intriguingly, echocardiography ruled out endocarditis, and there was no evidence of intraabdominal collections.

The patient was promptly admitted for further investigations and treatment. Blood cultures were obtained, which later confirmed S. Aureus bacteremia. This finding guided antibiotic therapy initiation, beginning with ceftriaxone and vancomycin. Given the positive blood cultures and the recommendations of infectious disease specialists, the antibiotic regimen was tailored to cefazolin to optimize treatment.

## 4 CONCLUSION AND RESULTS

Throughout his hospitalization, the patient encountered another challenge—peripheral line thrombophlebitis. During his hospitalization, the patient experienced another obstacle—peripheral line thrombophlebitis. Upon clinical review, the prevailing suspicion leaned towards septic tendencies rather than other hematological disorders, given the complex clinical manifestations observed.

Despite these complexities, the patient exhibited a remarkable response to treatment. He started to ambulate after 3 days of antibiotic therapy, and his clinical condition continued to improve. Blood cultures returned negative after 5 days of treatment, indicating a successful response to antibiotics. Importantly, the patient began maintaining adequate oxygen saturation on room air, signifying significant progress in his respiratory status.

## 5 DISCUSSION

This case of SPE originating from a small boil on the forehead is a rare and intriguing presentation, emphasizing the diverse nature of sources for embolic events. Our case serves as a reminder that unusual and seemingly minor foci of infection can also lead to significant pulmonary complications.

SPE is an uncommon clinical entity arising from the implantation of lytic pathogens mixed with fibrin into the pulmonary vasculature, leading to reinfection and embolization. The process involves the migration of microorganisms‐containing thrombi from the site of infection into the venous circulation, causing infarctions and micro‐abscesses within the pulmonary vascular beds and ultimately giving rise to SPE.^5^ SPE is characterized by imaging features such as 0.5–3.5 cm nodules, distinct vessels leading to nodules, and varying degrees of cavitation, primarily in the peripheral and lower lung fields. In our case, observations align with SPE, indicating multiple peripheral nodules and cavities. Cavity formation is attributed to embolism‐induced blood flow interruption, leading to tissue necrosis and infection.^6^

Additionally, the immune response and the presence of bacteria in the bloodstream activate the clotting system, potentially leading to thrombotic events. This hypercoagulable state may manifest as peripheral line thrombophlebitis, as observed in our case.

The prompt initiation of broad‐spectrum antibiotics was crucial in addressing the septicemia associated with Staphylococcus aureus infection. The subsequent adjustment of the antibiotic regimen based on blood culture results and infectious disease specialist recommendations reflects the importance of tailored therapy to optimize treatment outcomes.

Despite the challenges encountered, the patient's positive response to treatment is encouraging. The rapid improvement in respiratory status, along with negative blood cultures after 5 days of treatment, suggests successful management of the SPE. The demanding and fast‐paced environment of emergency medicine necessitates physicians to depend on innovative and modern technology to effectively fulfill their clinical responsibilities.^7^ This case highlights the importance of early diagnosis, appropriate antibiotic therapy, and vigilant management of associated complications in optimizing patient outcomes.

In summary, the progression from a small boil to SPE involves bacterial dissemination, septicemia, and the formation of septic emboli, ultimately affecting the lungs. The interplay between infection, inflammation, and the clotting system can contribute to thrombotic events in various parts of the body. Our case underscores the importance of promptly recognizing and treating even minor infections, particularly in individuals with underlying risk factors like poorly controlled diabetes.

## AUTHOR CONTRIBUTIONS

**Sanem Yildirim:** Data curation; investigation; methodology; writing – original draft. **Yavuz Yigit:** Formal analysis; methodology; supervision; writing – original draft; writing – review and editing. **Baha Hamdi Alkahlout:** Supervision; validation; writing – original draft; writing – review and editing. **Eslam Hussein Mohamed:** Methodology; writing – original draft. **Aftab Mohammad Azad:** Supervision; validation; writing – original draft; writing – review and editing.

## FUNDING INFORMATION

This research did not receive any specific funding.

## CONFLICT OF INTEREST STATEMENT

The authors declare no conflicts of interest.

## CONSENT

Written informed consent was obtained from the patient to publish this report in accordance with the journal's patient consent policy.

## Supporting information


Data S1.

## Data Availability

The corresponding author's data supporting this study's findings are available upon reasonable request.
