# Supplementary material for: Septic pulmonary embolism arising from a small temporal boil in a patient with diabetes mellitus type 2: A rare case report
Source: Clin Case Rep. 2024 May 10;12(5):e8790. doi: 10.1002/ccr3.8790 (PMC11087222; doi:10.1002/ccr3.8790)
Supplement: Supplementary file 1 — Data S1. [file CCR3-12-e8790-s001.docx]

**Answers to Reviewer Questions:**

**Question 1:** It is better to mention the results of the laboratory data you mentioned in the manuscript

**Answer:**

In the revised manuscript, we have incorporated the laboratory results ( Page 3, Line 86).

**Question 2:** Did you perform Doppler ultrasound on the neck vessels?

**Answer:**

We did not conduct a neck ultrasound for the patient and omitted this information in the text to maintain the flow. However, should the reviewers or editor prefer its inclusion, we would be more than willing to incorporate this detail into the manuscript.

**Question 3:** You mentioned that  "the patient encountered another challenge—peripheral line thrombophlebitis. This complication necessitated vigilant management and raised questions about potential thrombotic tendencies in the context of the infection", Did you do any excess investigations to assess his thrombotic tendencies

**Answer:**

We did not conduct additional investigations to assess thrombotic tendencies, and we have provided an explanation for this choice in the revised manuscript (Page 5,Line 124).

**Question 4:** It is better to mention his respiratory rate, heart rate, blood pressure, and oxygen saturation on admission to know the degree of severity of the patient.

**Answer:**

In the revised manuscript, we have incorporated the patient's vital signs upon admission (Page 3,Line 84).
